# Effects of the reaction temperature and Ba/Ti precursor ratio on the crystallite size of BaTiO_3_ in hydrothermal synthesis†

**DOI:** 10.1039/d2ra03707f

**Published:** 2022-09-29

**Authors:** Min Zhang, Joseph Falvey, Andrew L. Hector, Nuria Garcia-Araez

**Affiliations:** School of Chemistry, University of Southampton Highfield Southampton SO17 1BJ UK A.L.Hector@soton.ac.uk; The Faraday Institution, Quad One, Harwell Campus Didcot OX11 0RA UK

## Abstract

Nanocrystalline BaTiO_3_ has been prepared by hydrothermal synthesis from titanium isopropoxide and barium hydroxide octahydrate. Reaction conditions including synthesis temperature and Ba/Ti precursor ratio have been explored with the aim of producing BaTiO_3_ with small crystallites and a low concentration of defects. It has been found that the crystallite/particle size and tetragonality of the BaTiO_3_ samples increase as the synthesis temperature increases; and the crystallite/particle size of BaTiO_3_ is also affected by the Ba/Ti precursor ratio. The BaTiO_3_ sample synthesised using a Ba/Ti precursor ratio of 2 : 1 at a reaction temperature of 120 °C exhibited homogeneous crystallites of the smallest size of 107 nm. Additionally, the Ba/Ti precursor ratio of 2 : 1 with synthesis temperature of 220 °C was found to produce a smaller concentration of defects in BaTiO_3_.

## Abstract 2

Nanocrystalline BaTiO_3_ has been prepared *via* a hydrothermal synthesis. Reaction conditions including synthesis temperature and Ba/Ti precursor ratio/concentration have been systematically explored to produce small crystallites of phase-pure BaTiO_3_.

## Graphical abstract

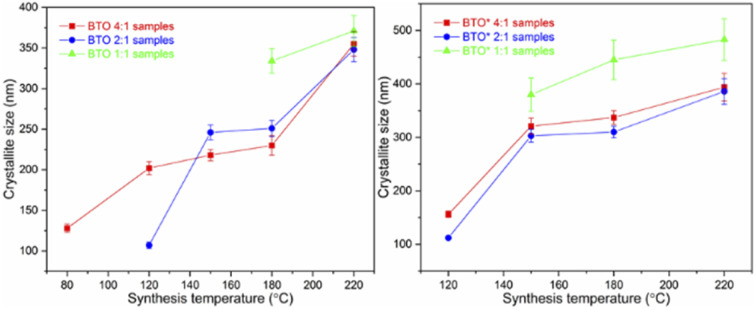

## 1. Introduction

Barium titanate (BaTiO_3_) is commonly used in multilayer capacitors, thermistors, transducers, electromechanical devices, electro-optical devices, dynamic random-access memory, field-effect transistors and actuators, *etc.*, due to its excellent dielectric and ferroelectric properties.^1^ BaTiO_3_ is both mechanically and chemically stable, and exhibits different crystalline structures depending on the temperature.^2,3^ Above 120 °C, BaTiO_3_ exhibits the classical perovskite structure with Ba^2+^ at the corners of the cubic unit cell, O^2−^ at the face centres and Ti^4+^ at the body centre; this structure is cubic and centrosymmetric and, therefore, the material does not exhibit a net dipole moment and hence behaves as a dielectric. Below 120 °C, BaTiO_3_ experiences a structural distortion: Ti^4+^ is displaced off its central position in the octahedron, in the direction of one of the O^2−^; this tetragonal distortion in the structure generates a net dipole moment, and hence the material behaves as a ferroelectric. BaTiO_3_ exhibits a very high dielectric constant, particularly at temperatures close to the ferroelectric–dielectric transition.

The overriding goal in BaTiO_3_ synthesis in recent years is to create smaller and more uniform particles to allow for thinner layers of the ceramic material to be used in electronic device components such as capacitors.^4^ Therefore it is important to synthesise BaTiO_3_ crystallites of the smallest size possible. Manufacturers of multilayer capacitors use BaTiO_3_ because of its excellent dielectric properties, reliability, high breakdown voltage and low cost of production.^5^ Other uses for BaTiO_3_ in the electronics industry include temperature sensing (temperature trips), overcurrent protection (self-resetting fuses) and electronic timing circuitry. These devices use the positive temperature coefficient of resistivity (PTCR) property in BaTiO_3_ materials to control the resistance in a circuit as a function of temperature in the form of thermistors.^6,7^ This PTCR effect has also recently been shown to be able to prevent thermal runaway in batteries.^8^

There are multiple synthesis methods used in industry to produce BaTiO_3_, however manufacturers of multilayer capacitors and PTCR devices have specific requirements for the ceramic material if it is to be used in the preparation of dielectric layers. The method must produce a high purity product with homogeneous composition to ensure stable and consistent dielectric properties. A route that causes strong agglomeration will result in a non-uniform particle size, which is problematic. For the production of multilayer capacitors, in general, solution-based techniques such as hydrothermal synthesis are used to prepare very fine particles, as these techniques achieve a homogeneous, phase-pure stoichiometric BaTiO_3_ with synthesis temperatures far lower than solid state methods (>600 °C).^5,9^ Nanosized particles of BaTiO_3_ can be achieved by controlling reaction conditions such as synthesis temperature and time, Ba and Ti precursor categories and Ba/Ti precursor ratio.^4^ Zeng *et al.* prepared nanocrystalline BaTiO_3_ from barium hydroxide octahydrate and titanium isopropoxide at low temperature (<100 °C) and ambient pressure, and reported the impact of the reaction temperature and Ba/Ti precursor ratio on the morphology and size of BaTiO_3_.^10,11^ Then, Nahm *et al.* used the same precursors and hydrothermal conditions for the synthesis of nanocrystalline BaTiO_3_, thus producing a one-step reaction process with faster reaction times, but they only employed one temperature (220 °C) and Ba/Ti precursor ratio (4 : 1).^12^ In this work, we employ the same precursors and hydrothermal conditions for the synthesis of nanocrystalline BaTiO_3_, but we systematically explore the effect of the reaction temperature (from 80 to 220 °C) and Ba/Ti precursor ratio (from 4 : 1 to 1 : 1), and we characterise the reaction product to identify the optimal reaction conditions in terms of small particle size and low concentration of defects.

## 2. Experimental

### 2.1. Materials synthesis

**Scheme 1:**
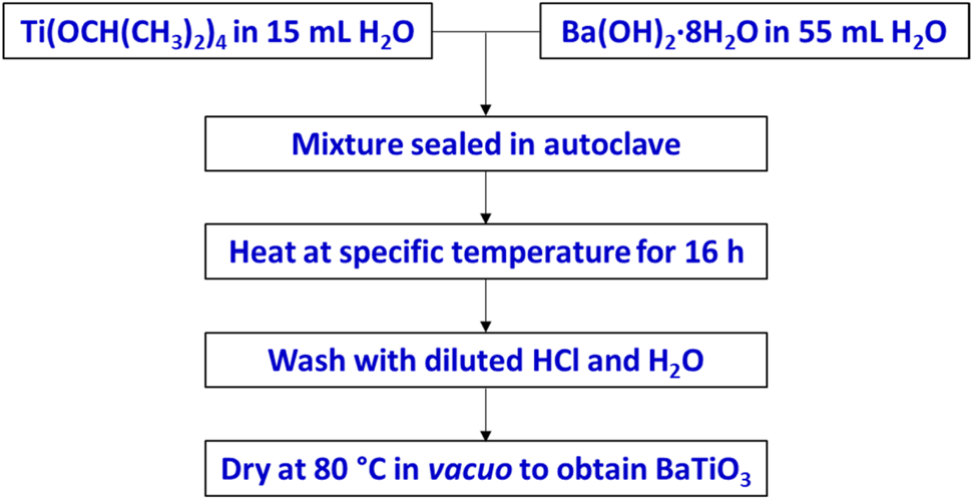
Hydrothermal synthesis to prepare BaTiO_3_, using various Ba/Ti precursor ratios/concentrations and reaction temperatures.

The BaTiO_3_ samples were synthesized *via* a hydrothermal method,^12^ as illustrated in Scheme 1. As a Ti precursor, the Ti(OCH(CH_3_)_2_)_4_ (≥97% purity, Sigma Aldrich) was added dropwise to 15 mL of ice cold distilled water while being constantly stirred. Upon addition of the Ti(OCH(CH_3_)_2_)_4_, the distilled water became turbid then a white precipitate formed. The reaction mixture was stirred in ice bath for 1 h then at room temperature for a further 2 h to allow the reaction to reach completion. To make up the Ba precursor, an appropriate amount of Ba(OH)_2_·8H_2_O (≥98% purity, Emsure) was dissolved in 55 mL of distilled water. The exact concentration of each precursor used is specific to the reaction parameter being investigated and is specified in the results and discussion section. The Ba precursor solution was added to the Ti precursor suspension to make up the reaction mixture. The reaction mixture was then transferred to an autoclave where it was sealed and heated. Each reaction mixture was heated at a specific temperature in an oven for 16 h. The white solid that formed in the reaction was collected and washed in a centrifuge at 4500 rpm for 8 min. The solid was washed with diluted HCl once, then with distilled water 3 times. After that, the solid was dried at 80 °C for 12 h in a vacuum oven to obtain BaTiO_3_ samples.

### 2.2. Materials characterisation

X-ray diffraction (XRD) spectra were collected using a Bruker D2 Phaser with Cu-Kα radiation. Rietveld analysis was performed using the GSAS package.^13^ The crystallite sizes were calculated from the Lorentzian broadening components collected from the Rietveld fits to the XRD patterns, according to 
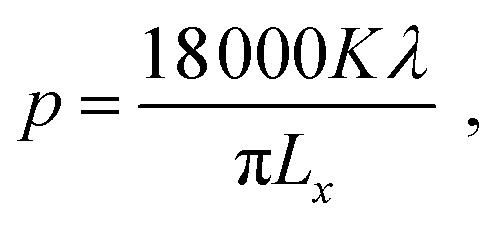
 where *p* is the crystallite size (unit: nm), *K* is the Scherrer constant, *λ* is the wavelength of the incident X-rays (unit: nm), and *L*_*x*_ is the refined Lorentzian value.^13^ Scanning electron microscopy (SEM) micrographs were collected on a JEOL JSM-6500F operated at 10 kV, and images were analysed using the ImageJ software and Gaussian function. Transmission electron microscopy (TEM) used a Hitachi HT7700 (120 kV). Raman spectra were recorded using a Renishaw inVia confocal microscope, and the spectra were analysed using the WiRE 4.1 software. Dynamic light scattering (DLS) measurements were carried out with a Malvern Zetasizer Nano ZS, and three sequential measurements were performed for each sample. Wavelength dispersive X-ray analysis (WDS) used a Thermofisher Magnaray detector with Noran System 7 processing. Thermogravimetric analysis (TGA) was carried out using a NETZSCH TG 209 F1 Libra instrument, with a heating process of at 1 °C min^−1^ from 25 to 200 °C, then at 10 °C min^−1^ from 200 to 1000 °C under Ar atmosphere.

## 3. Results and discussion

### 3.1. Effects of the reaction temperature on the crystallite size, morphology and composition of BaTiO_3_ in hydrothermal synthesis

Table 1 summarises the experimental conditions that successfully led to the production of BaTiO_3_. Using a constant Ti precursor concentration of 0.045 mol L^−1^ and the Ba precursor concentration was varied from 0.045 to 0.18 mol L^−1^, thus Ba/Ti precursor ratios of 1 : 1, 2 : 1 and 4 : 1 have been achieved. These three reaction mixtures were heated at various temperatures of 80, 120, 150, 180 and 220 °C to obtain different BaTiO_3_ samples.

**Table 1:** The variations of Ba precursor concentrations, Ba/Ti precursor ratios and reaction temperatures, with Ti precursor concentration kept constant at 0.045 mol L^−1^ in hydrothermal syntheses of BaTiO_3_ samples; crystallite sizes obtained from the Rietveld fits to the XRD patterns^a^

Sample	Ba precursor concentration (mol L^−1^)	Ba/Ti precursor ratio	Synthesis temperature (°C)	Crystallite size (nm)
BTO 4 : 1 – 80 °C	0.18	4 : 1	80	128 (5)
BTO 4 : 1 – 120 °C			120	202 (8)
BTO 4 : 1 – 150 °C			150	218 (7)
BTO 4 : 1 – 180 °C			180	230 (12)
BTO 4 : 1 – 220 °C			220	355 (15)

BTO 2 : 1 – 120 °C	0.09	2 : 1	120	107 (4)
BTO 2 : 1 – 150 °C			150	246 (9)
BTO 2 : 1 – 180 °C			180	251 (10)
BTO 2 : 1 – 220 °C			220	348 (15)

BTO 1 : 1 – 180 °C	0.045	1 : 1	180	334 (15)
BTO 1 : 1 – 220 °C			220	371 (19)

a Note that samples synthesized using Ba/Ti precursor ratio of 1 : 1 at 80, 120 and 150 °C (as well as using 2 : 1 ratio at 80 °C) had very low yields, thus no further investigation proceeded.

**Fig. 1:**
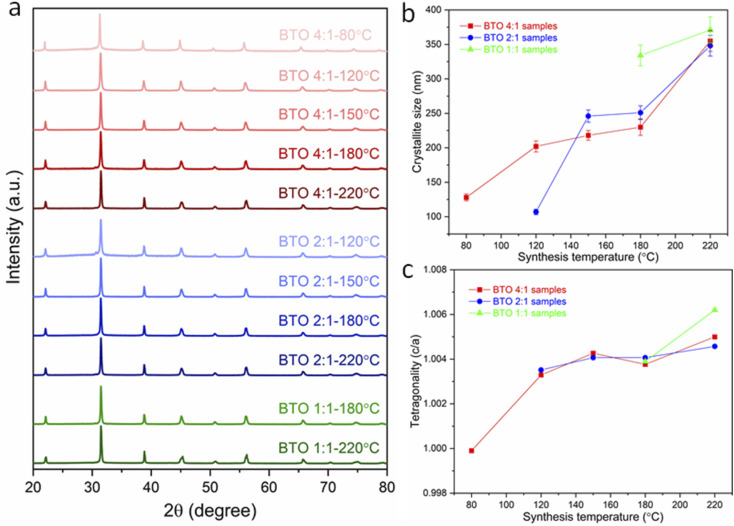
(a) XRD patterns of BaTiO_3_ samples hydrothermally synthesised with Ba/Ti precursor ratios of 4 : 1, 2 : 1 and 1 : 1, at various reaction temperatures of 80, 120, 150, 180 and 220 °C; (b) crystallite size and (c) tetragonality (*c*/*a*) of BaTiO_3_ samples plotted against reaction temperature in hydrothermal synthesis using Ba/Ti precursor ratios of 4 : 1, 2 : 1 and 1 : 1, obtained from the Rietveld fits to the XRD patterns (sample labels explained in Table 1).

The XRD patterns of the obtained BaTiO_3_ samples (Fig. 1a) indicate that phase-pure BaTiO_3_ was obtained in all cases, since no impurity phases were detected. Habib *et al.* detected the formation of a BaCO_3_ impurity for BaTiO_3_ synthesised hydrothermally, since a BaCO_3_ peak was observed at a 2*θ* value of approximately 24°.^14^ BaCO_3_ forms in the heating step in a reaction between Ba^2+^ ions in the solution and any CO_2_ that may be in the air space above the reaction mixture. In our work, the BaTiO_3_ samples were washed with diluted HCl, which would remove BaCO_3_ impurities, and indeed, the XRD data do not show the presence of any BaCO_3_. The XRD results also do not show the presence of any unreacted rutile or anatase TiO_2_. While some previous work employed TiO_2_ as precursor for the BaTiO_3_ hydrothermal synthesis,^14,15^ in this work, a metal–organic [Ti(OCH(CH_3_)_2_)_4_] with higher reactivity is used as Ti precursor. This precipitates as amorphous TiO_2_ (Fig. S1†) in water improving the likelihood that it will fully react with the Ba precursor in hydrothermal synthesis, such that any unreacted TiO_2_ may not be observed.

From the Lorentzian values collected from the Rietveld fits (Fig. S2†) to the XRD patterns, the crystallite sizes of all samples have been calculated and presented in Table 1. The obtained lattice parameters (*a* and *c*), tetragonality (*c*/*a*), Lorentzian values (*L*_*x*_ and *L*_*y*_) and reliability factors (*R*_wp_ and *R*_p_) are listed in Table S1.†Fig. 1b shows the variation of the crystallite size of BaTiO_3_*versus* the reaction temperature in hydrothermal synthesis with Ba/Ti precursor ratios of 4 : 1, 2 : 1 and 1 : 1. In all three Ba/Ti precursor ratios, there is a general decrease in crystallite size with decreasing synthesis temperature. Using a 4 : 1 Ba/Ti precursor ratio, the crystallite size of the obtained BaTiO_3_ reduces from 355 nm at 220 °C to 128 nm at 80 °C. At a 2 : 1 ratio the crystallite size falls from 348 nm at 220 °C to 107 nm at 120 °C. The 1 : 1 ratio shows a crystallite size that drops from 371 nm at 220 °C to 334 nm at 180 °C. The synthesis temperature has been found to have a profound effect on the crystallite size of the BaTiO_3_ samples with a lower synthesis temperature producing crystallites of a smaller size. The strong growth of BaTiO_3_ crystallites to their final size is typical of an Ostwald ripening process which involves the growth of large particles at the expense of smaller particles. An accelerated Ostwald ripening process with increasing temperature is likely to be the cause of the increase in crystallite size.^15–17^ BaTiO_3_ with crystallites as small as 107 nm have been produced using a 2 : 1 Ba/Ti precursor ratio and reacted at 120 °C.

The tetragonality (*c*/*a*) has been used to denote the degree of tetragonal–cubic distortion,^18^ and BaTiO_3_ samples with large tetragonality are essential to obtain multilayer capacitors with large capacitances.^12^Fig. 1c presents the tetragonality variation of BaTiO_3_ against the reaction temperature in hydrothermal synthesis with Ba/Ti precursor ratios of 4 : 1, 2 : 1 and 1 : 1. The increasing *c*/*a* value suggests that the tetragonality of the crystal structure increases with the increasing synthesis temperature in all three Ba/Ti precursor ratios. In general, the tetragonality of the BaTiO_3_ is reduced by the presence of hydroxyl defects in the product.^19^ Therefore, the BaTiO_3_ samples synthesised at higher temperatures are considered to contain less hydroxyl defects. This can be confirmed by the TGA characterisation presented below.

**Fig. 2:**
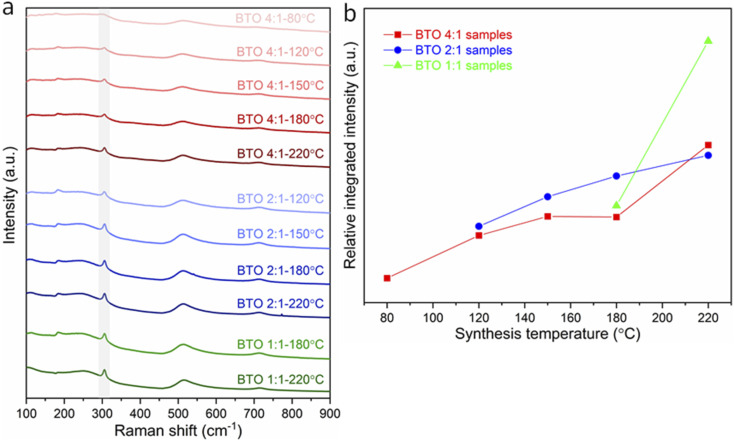
(a) Raman spectra of BaTiO_3_ samples hydrothermally synthesised with Ba/Ti precursor ratios of 4 : 1, 2 : 1 and 1 : 1, at various reaction temperatures of 80, 120, 150, 180 and 220 °C (peaks at ∼307 cm^−1^ being marked as shaded); (b) relative integrated intensities of the Raman peak at 307 cm^−1^ for BaTiO_3_ samples plotted against reaction temperature in hydrothermal synthesis using Ba/Ti precursor ratios of 4 : 1, 2 : 1 and 1 : 1 (sample labels explained in Table 1).

The variations in tetragonality of hydrothermally synthesized BaTiO_3_ samples can also be confirmed by Raman characterisation. As shown in Fig. 2a, the Raman spectrum of tetragonal BaTiO_3_ presents the Raman active modes A1 (TO), E (TO) at ∼180 cm^−1^, A1 (TO) at ∼270 cm^−1^, B1, E (LO + TO) at ∼307 cm^−1^, A1 (TO), E (TO) at ∼515 cm^−1^ and A1 (LO), E (LO) at ∼720 cm^−1^.^18^ It has been reported that the sharp peak at ∼307 cm^−1^ (shaded in Fig. 2a) is particularly sensitive to the tetragonality of the crystal structure, with its intensity increasing when BaTiO_3_ becomes more tetragonal.^20–22^Fig. 2b shows the variation of the relative integrated intensity of the peak at 307 cm^−1^ for BaTiO_3_*versus* the reaction temperature in hydrothermal synthesis with Ba/Ti precursor ratios of 4 : 1, 2 : 1 and 1 : 1. The increasing integrated intensity of the peak at 307 cm^−1^ suggests that the tetragonality of the crystal structure increases with the increasing synthesis temperature in all three Ba/Ti precursor ratios. This trend is consistent with the results obtained from the XRD Rietveld analysis.

**Fig. 3:**
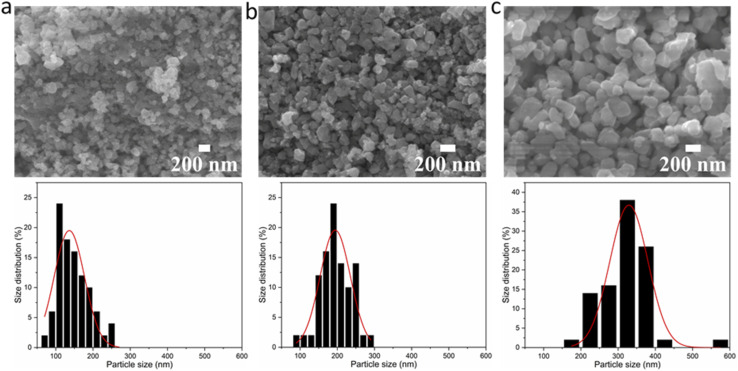
SEM images of BaTiO_3_ samples hydrothermally synthesised using a 4 : 1 Ba/Ti precursor ratio with reaction temperatures of (a) 80 °C, (b) 150 °C and (c) 220 °C; the patterns below to show the grain sizes of the samples determined using the ImageJ software, and the size distributions analysed with a Gaussian function.

**Fig. 4:**
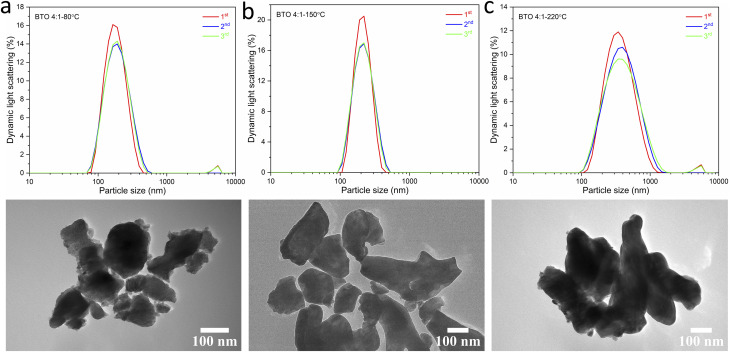
DLS patterns (above) and TEM images (below) of BaTiO_3_ samples hydrothermally synthesised using a 4 : 1 Ba/Ti precursor ratio with reaction temperatures of (a) 80 °C, (b) 150 °C and (c) 220 °C.

To investigate any trends between the size and morphology of individual particles and the reaction temperature, Fig. 3 shows the SEM images and particle size distributions of BaTiO_3_ samples synthesised using a 4 : 1 Ba/Ti precursor ratio with reaction temperatures of 80, 150 and 220 °C in hydrothermal synthesis. The particle sizes of BaTiO_3_ samples were determined from analysing SEM images using the ImageJ software, and the size distributions were obtained by fitting with a Gaussian function. The BaTiO_3_ sample synthesised at 80 °C shows the smallest average particle size of 136 nm with ill-defined morphology, and the small particles agglomerated into clusters. As the synthesis temperature increases to 150 and 220 °C, the average particle size increases to 194 and 329 nm, respectively, and the edges of particles become well-defined. This can be confirmed by the DLS and TEM characterisations shown in Fig. 4. The average particle sizes obtained from the DLS measurements are 176, 203 and 335 nm for BaTiO_3_ samples synthesised at 80, 150 and 220 °C, respectively. Note that the smaller particles are more likely to agglomerate into clusters, thus the samples with larger particle sizes gave better accuracy during DLS measurements. According to the SEM, TEM and DLS characterisations, it can be concluded that the BaTiO_3_ particle size increases as the synthesis temperature is increased. This trend agrees with the result obtained from the Rietveld fits to the XRD data.

**Fig. 5:**
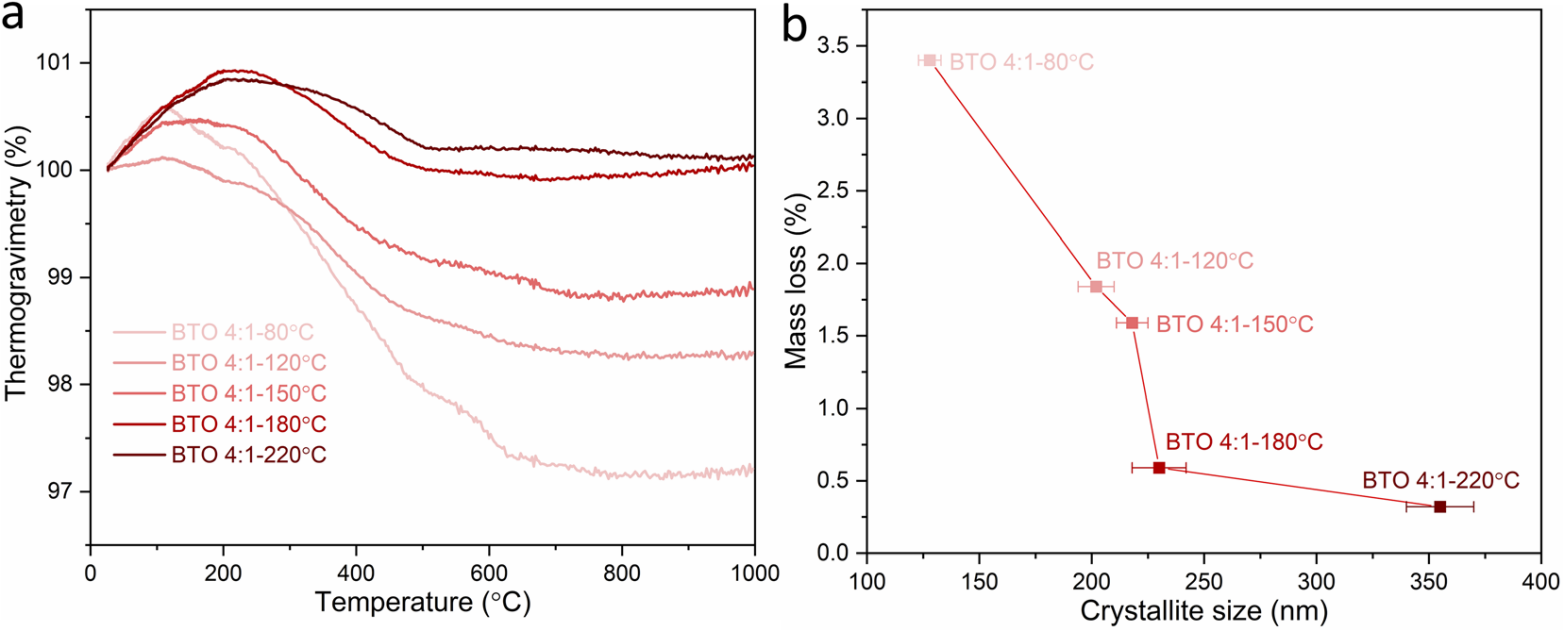
(a) TGA curves and (b) mass losses against crystallite sizes of BaTiO_3_ samples hydrothermally synthesised using a 4 : 1 Ba/Ti precursor ratio with reaction temperatures of 80, 120, 150, 180 and 220 °C, examined by heating in Ar with a heating process of at 1 °C min^−1^ from 25 to 200 °C, then at 10 °C min^−1^ from 200 to 1000 °C (sample labels explained in Table 1).

TGA was carried out for BaTiO_3_ samples hydrothermally synthesised using a 4 : 1 Ba/Ti precursor ratio with reaction temperatures of 80, 120, 150, 180 and 220 °C. Fig. 5a and Table 2 show the mass losses of the BaTiO_3_ samples in the TGA characterisations as the temperature is raised from 25 to 1000 °C in an inert Ar atmosphere. For the BaTiO_3_ samples that have been synthesised at 220, 180, 150, 120 and 80 °C, there are increasing mass losses of 0.3%, 0.6%, 1.6%, 1.8% and 3.4%, respectively, with the mass change attributed to loss of surface hydroxyl groups in the temperature range of 100–800 °C.^23^ The TGA analysis indicates that as the synthesis temperature of BaTiO_3_ decreases, there is a greater amount of mass lost when the BaTiO_3_ product is heated.

**Table 2:** Mass losses of BaTiO_3_ samples hydrothermally synthesised using a 4 : 1 Ba/Ti precursor ratio with reaction temperatures of 80, 120, 150, 180 and 220 °C, for the calculation of *x* in the form of 
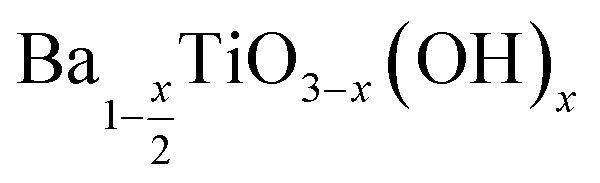

Synthesis temperature (°C)	Mass loss (%)	Value of *x*
220	0.3	0.08
180	0.6	0.15
150	1.6	0.37
120	1.8	0.41
80	3.4	0.70

The increase in surface hydroxyl defects can be linked to the increased surface area of crystallites when formed at lower temperatures.^24^Table 2 shows the corrected stoichiometric formulas of the samples with the removal of hydroxyl defects in the form of 
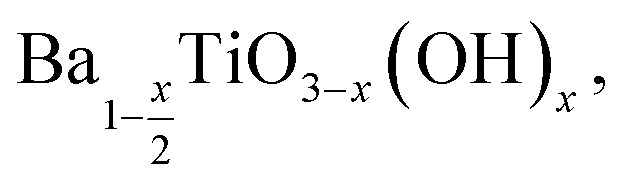
 according to 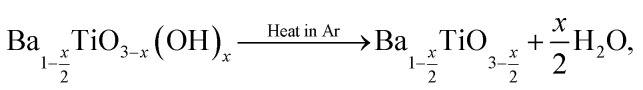 which exhibits increasing *x* values as the synthesis temperature decreases. The BaTiO_3_ samples synthesised using a 4 : 1 Ba/Ti precursor ratio with decreasing reaction temperatures of 220, 180, 150, 120 and 80 °C exhibit decreasing crystallite sizes of 355, 230, 218, 202 and 128 nm, respectively. Decreasing the synthesis temperature results in a smaller crystallite size of BaTiO_3_ and a larger surface area resulting in a greater number of hydroxyl defects in the product, and therefore a greater mass loss when heated in the TGA characterisation, as shown in Fig. 5b.

### 3.2. Effects of the Ba/Ti precursor ratio/concentration on the crystallite size, morphology and composition of BaTiO_3_ in hydrothermal synthesis

As shown in Table 1, three different Ba/Ti precursor ratios (1 : 1, 2 : 1 and 4 : 1) were employed in this work, and Fig. 1b shows that samples synthesised using Ba/Ti precursor ratios of 4 : 1 and 2 : 1 exhibit crystallites of smaller sizes, as obtained from the Rietveld fits to the XRD data, than those using a Ba/Ti precursor ratio of 1 : 1.

**Fig. 6:**
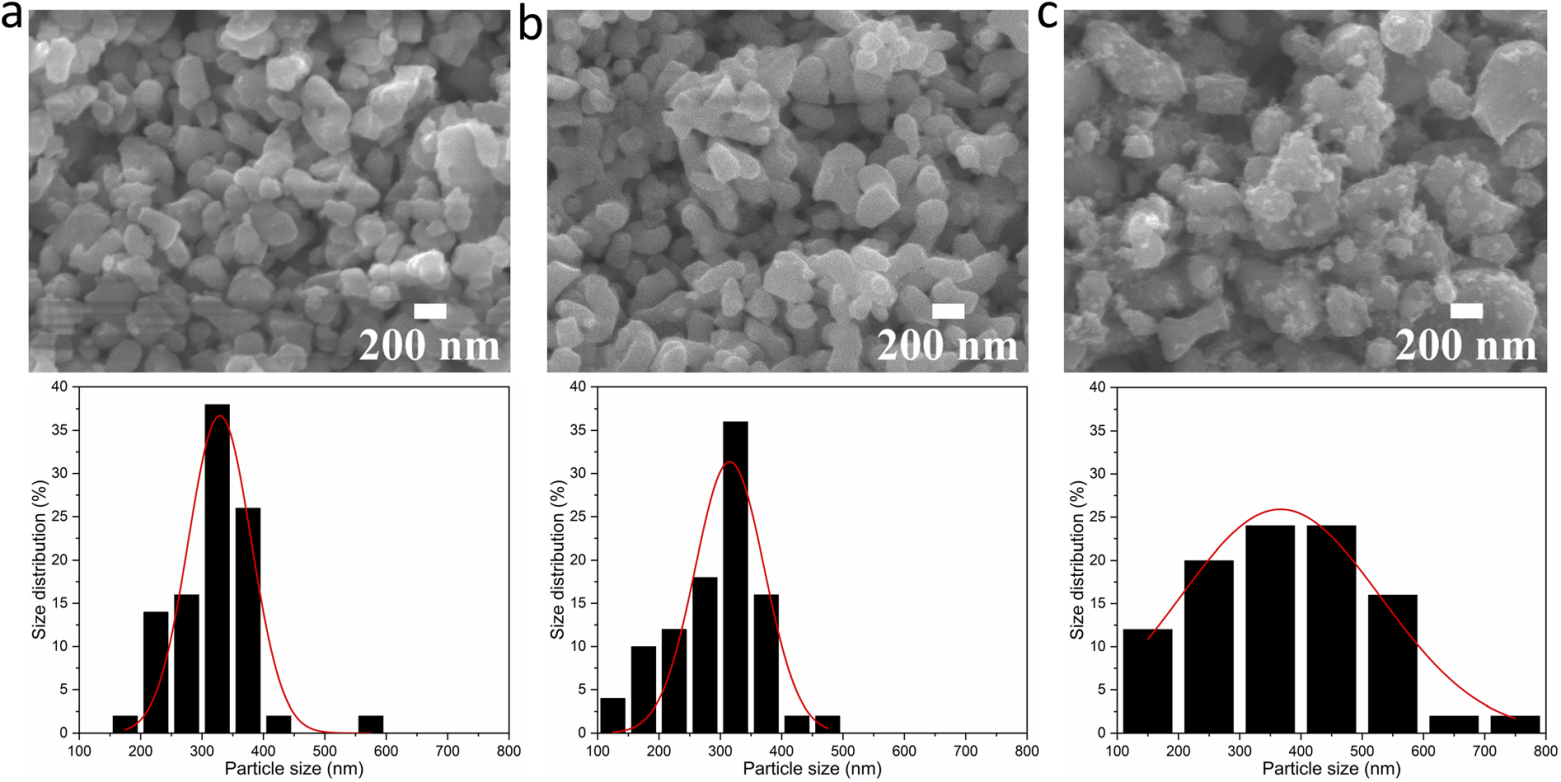
SEM images of BaTiO_3_ samples hydrothermally synthesised at 220 °C using Ba/Ti precursor ratios of (a) 4 : 1, (b) 2 : 1 and (c) 1 : 1; the patterns below to show the grain sizes of the samples determined using the ImageJ software, and the size distributions analysed with a Gaussian function.

**Fig. 7:**
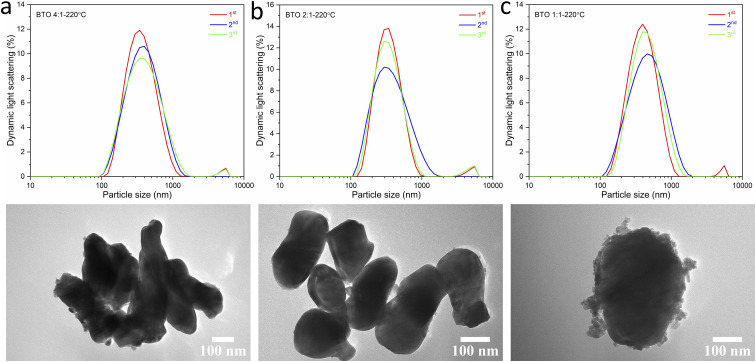
DLS patterns (above) and TEM images (below) of BaTiO_3_ samples hydrothermally synthesised at 220 °C using Ba/Ti precursor ratios of (a) 4 : 1, (b) 2 : 1 and (c) 1 : 1.

This result is supported by the SEM images of the BaTiO_3_ samples, and the particle size distributions obtained from the SEM images, as shown in Fig. 6. The BaTiO_3_ sample synthesised using Ba/Ti precursor ratios of 4 : 1 and 2 : 1, at 220 °C, exhibit average particle sizes of 329 and 315 nm, respectively. With the Ba/Ti precursor ratio of 1 : 1, the obtained BaTiO_3_ sample shows a larger average particle size of 367 nm and a wider size distribution. The particles are also less elliptical and much more irregular in shape with a more agglomerated structure. These results are corresponding with the results obtained from the DLS and TEM characterisations, as shown in Fig. 7. The average particle sizes obtained from the DLS measurements are 335, 324 and 379 nm for BaTiO_3_ samples synthesised using Ba/Ti precursor ratios of 4 : 1, 2 : 1 and 1 : 1, respectively.

The larger and more irregular particle size of BaTiO_3_ for the lower Ba/Ti precursor ratio of 1 : 1 can be understood by realising that the lower the concentration of Ba precursor in the initial reaction mixture will produce a lower number of nucleation points where the growth of BaTiO_3_ is initiated. Eckert *et al.* studied the mechanism for the hydrothermal formation of BaTiO_3_ from barium hydroxide octahydrate and anatase titania,^25^ and concluded that the growth of BaTiO_3_ particles is much slower than the formation of nuclei, however by reducing the concentration of Ba^2+^ in the reaction mixture, the rate of the particle growth could begin to compete with the rate of initial nuclei formation, leading to an uneven size distribution in the final BaTiO_3_ product, such as that seen in Fig. 6c.

In summary, the XRD, SEM, TEM and DLS results consistently show that the crystallite size and morphology of BaTiO_3_ are affected by the Ba precursor concentration and Ba/Ti precursor ratio in hydrothermal synthesis, and the BaTiO_3_ samples synthesised using Ba/Ti precursor ratios of 2 : 1 and 4 : 1 exhibit homogeneous crystallites with smaller sizes.

**Fig. 8:**
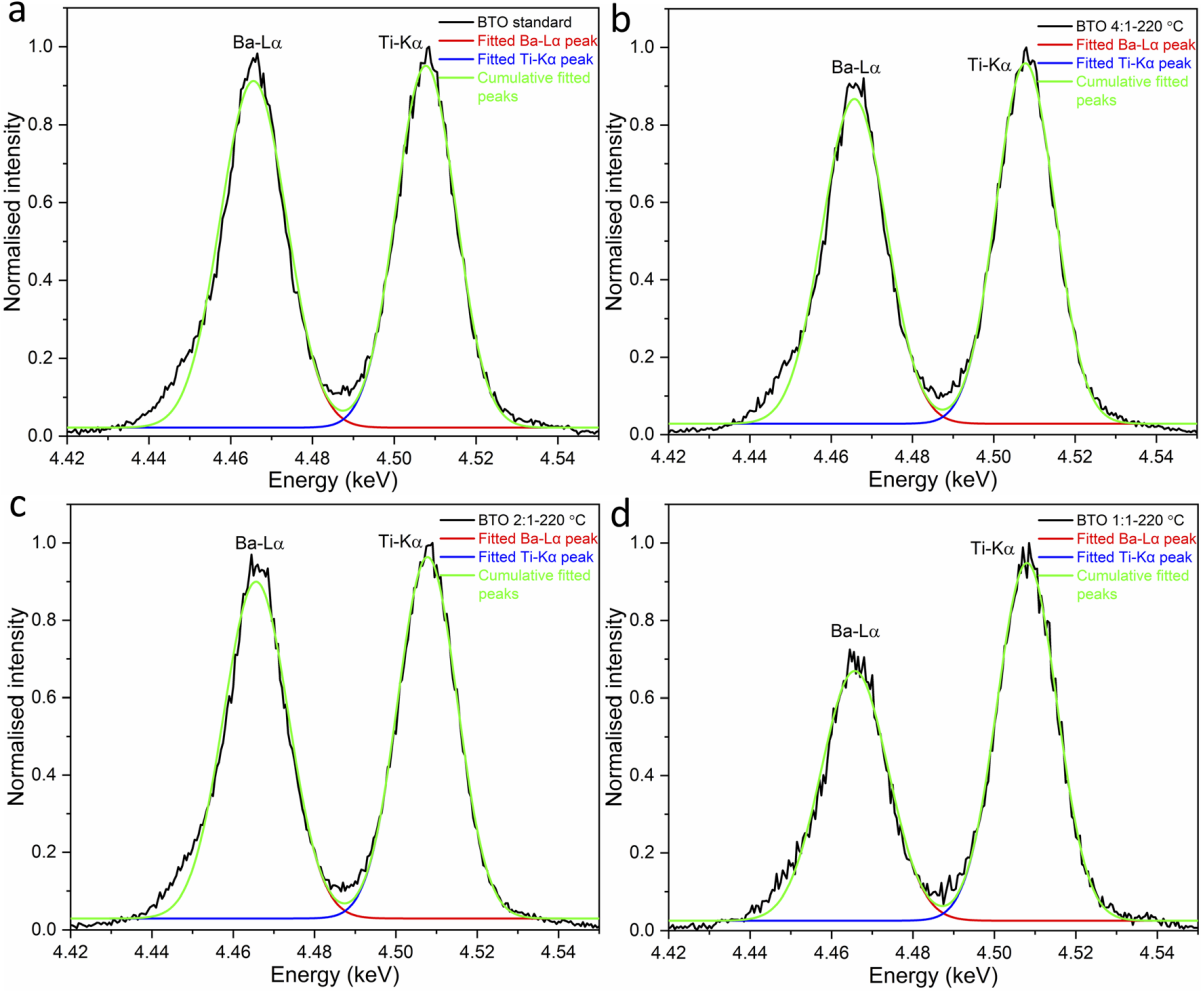
Fit of the WDS spectra peaks for the Ba–L_α_ and Ti–K_α_ peaks of (a) the standard BaTiO_3_ and BaTiO_3_ samples hydrothermally synthesised at 220 °C using Ba/Ti precursor ratios of (b) 4 : 1, (c) 2 : 1 and (d) 1 : 1 (sample labels explained in Table 1).

WDS analysis was carried out on the standard BaTiO_3_ and BaTiO_3_ samples hydrothermally synthesised at 220 °C using Ba/Ti precursor ratios of 4 : 1, 2 : 1 and 1 : 1 (Fig. S3†). The Ba–L_α_ and Ti–K_α_ peaks overlapped strongly in a standard energy dispersive X-ray spectroscopy (EDS). With WDS they were successfully resolved, however some degree of overlap remained. To determine the areas under the two separate peaks, they were fitted using Origin software (Fig. 8). The peak areas of the synthesised BaTiO_3_ samples were compared to a standard BaTiO_3_ sample made by solid-state synthesis which is known to have a Ba/Ti ratio of 1 : 1. The peak areas for the Ba-L_α_ and Ti-K_α_ peaks for the WDS spectra and the calculated stoichiometric Ba/Ti ratios of the BaTiO_3_ samples are presented in Table 3.

**Table 3:** Analysis of peak areas for the Ba–L_α_ and Ti–K_α_ peaks for the WDS spectra and the calculated stoichiometric Ba/Ti ratios of the standard BaTiO_3_ and BaTiO_3_ samples hydrothermally synthesised at 220 °C using Ba/Ti precursor ratios of 4 : 1, 2 : 1 and 1 : 1

Ba/Ti precursor ratio	Area of Ba-L_α_ peak	Area of Ti–K_α_ peak	Stoichiometric Ba/Ti ratio in BaTiO_3_
Standard	0.01806	0.01709	1 : 1
4 : 1	0.01658	0.01684	0.93 : 1
2 : 1	0.01735	0.01705	0.96 : 1
1 : 1	0.01324	0.01703	0.74 : 1

As shown in Table 3, all hydrothermally synthesised samples are Ba deficient. This is to be expected as the acid washing step in the synthesis has the ability to leach Ba^2+^ from the surface of the particles.^7^ In addition, Ba^2+^ vacancies in the sample also occur to maintain electroneutrality when hydroxyl defects are present as described above.^19^ The sample synthesised using a Ba/Ti precursor ratio of 1 : 1 exhibits a far lower Ba content in the product with a stoichiometric Ba/Ti ratio of 0.74 : 1. Raising the Ba/Ti precursor ratios to 2 : 1 and 4 : 1 increases the stoichiometric Ba/Ti ratios in the products to 0.96 : 1 and 0.93 : 1, respectively.

### 3.3. Effects of the Ti precursor concentration on the crystallite size of BaTiO_3_ in hydrothermal synthesis

Table 4 shows a second set of experimental conditions that successfully led to the production of BaTiO_3_. In this case, the Ba precursor concentration was kept constant at 0.09 mol L^−1^, while the Ti precursor concentration was varied from 0.0225 to 0.09 mol L^−1^, thus giving Ba/Ti precursor ratios of 4 : 1, 2 : 1 and 1 : 1. Note that the Ba/Ti precursor ratios are the same as for the first set of experiments, described in Table 1, and the reaction temperatures were also the same.

**Table 4:** The variations of Ti precursor concentrations, Ba/Ti precursor ratios and reaction temperatures, with Ba precursor concentration kept constant at 0.09 mol L^−1^ in hydrothermal syntheses of BaTiO_3_ samples; crystallite sizes obtained from the Rietveld fits to the XRD patterns^a^

Sample	Ti precursor concentration (mol L^−1^)	Ba/Ti precursor ratio	Synthesis temperature (°C)	Crystallite size (nm)
BTO* 4 : 1–120 °C	0.0225	4 : 1	120	156 (6)
BTO* 4 : 1 – 150 °C			150	321 (15)
BTO* 4 : 1 – 180 °C			180	337 (13)
BTO* 4 : 1 – 220 °C			220	394 (26)

BTO* 2 : 1 – 120 °C	0.045	2 : 1	120	112 (3)
BTO* 2 : 1 – 150 °C			150	303 (12)
BTO* 2 : 1 – 180 °C			180	310 (11)
BTO* 2 : 1 – 220 °C			220	386 (24)

BTO* 1 : 1 – 150 °C	0.09	1 : 1	150	380 (31)
BTO* 1 : 1 – 180 °C			180	445 (37)
BTO* 1 : 1 – 220 °C			220	483 (39)

a Note that samples synthesized using Ba/Ti precursor ratio of 1 : 1 at 80 and 120 °C (as well as using 2 : 1 and 4 : 1 ratio at 80 °C) had very low yields, thus no further investigation proceeded.

**Fig. 9:**
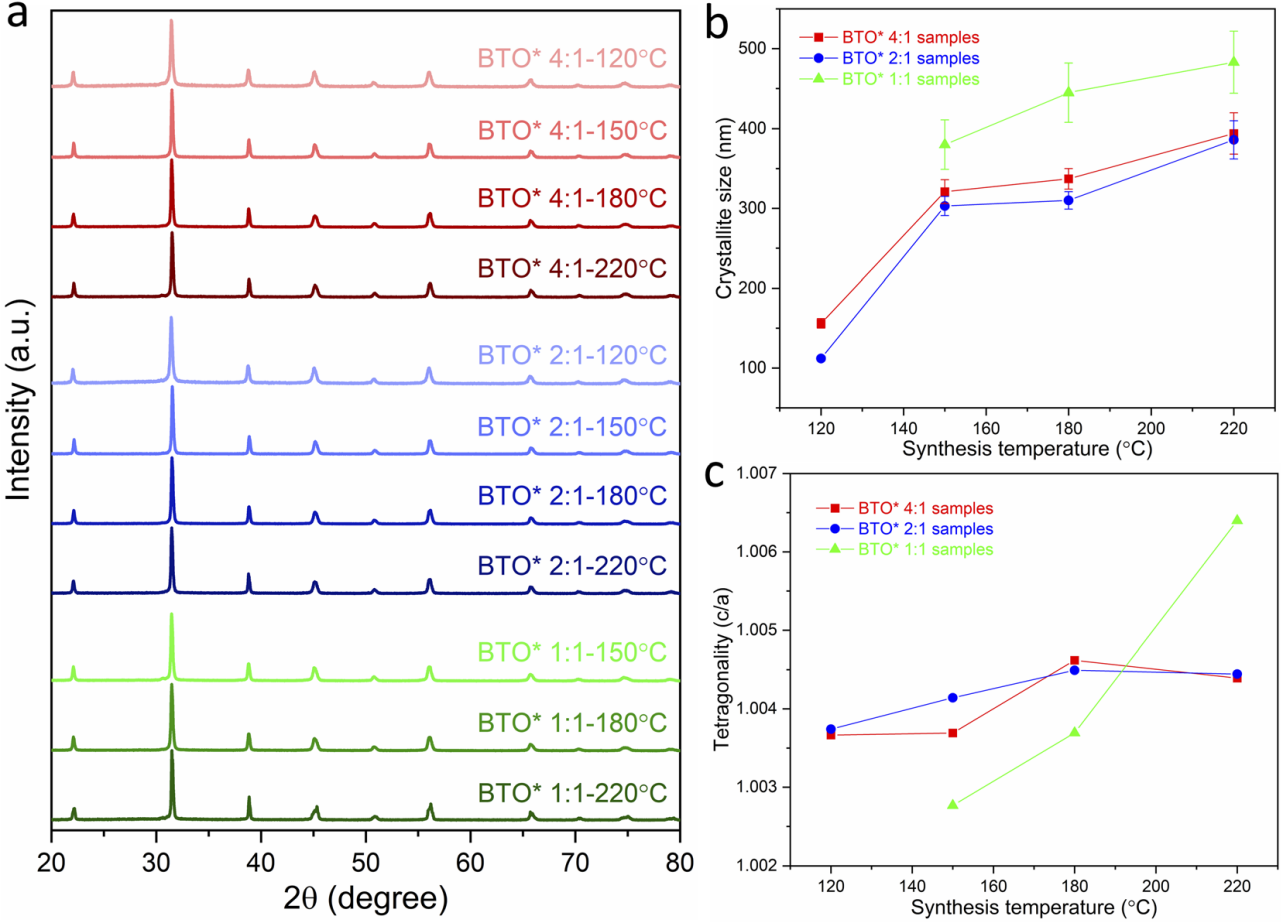
(a) XRD patterns of BaTiO_3_ samples hydrothermally synthesised using Ba/Ti precursor ratios of 4 : 1, 2 : 1 and 1 : 1 with Ba precursor concentration kept constant at 0.09 mol L^−1^, and reacted at various synthesis temperatures of 120, 150, 180 and 220 °C; (b) crystallite size and (c) tetragonality (*c*/*a*) of BaTiO_3_ samples plotted against reaction temperature in hydrothermal synthesis using Ba/Ti precursor ratios of 4 : 1, 2 : 1 and 1 : 1, obtained from the Rietveld fits to the XRD patterns (sample labels explained in Table 4).

As before for the previous set of conditions, the XRD patterns of the obtained BaTiO_3_ samples (Fig. 9a) indicate that phase-pure BaTiO_3_ was prepared in all cases, with no impurity phases detected. This can be attributed to the diluted acid wash step in the hydrothermal synthesis to remove any BaCO_3_ impurities that may have formed in the reaction, as well as to the use of a metal–organic [Ti(OCH(CH_3_)_2_)_4_] as the Ti precursor to precipitate amorphous TiO_2_ with higher reactivity in hydrothermal synthesis to avoid any unreacted TiO_2_ impurities.^14,15^

From the Lorentzian values collected from the Rietveld fits (Fig. S4†) to the XRD patterns, the crystallite sizes of all samples have been calculated and presented in Table 4. The obtained lattice parameters (*a* and *c*), tetragonality (*c*/*a*), Lorentzian values (*L*_*x*_ and *L*_*y*_) and reliability factors (*R*_wp_ and *R*_p_) are listed in Table S2.†Fig. 9b shows the variation of the crystallite size of BaTiO_3_*versus* the reaction temperature in hydrothermal synthesis with Ba/Ti precursor ratios of 4 : 1, 2 : 1 and 1 : 1. Similar to the trend observed in Fig. 1b, in all three Ba/Ti precursor ratios, there is a general decrease in crystallite size with decreasing synthesis temperature. Using a 4 : 1 Ba/Ti precursor ratio, the crystallite size of the obtained BaTiO_3_ reduces from 394 nm at 220 °C to 156 nm at 120 °C. At a 2 : 1 ratio the crystallite size falls from 386 nm at 220 °C to 112 nm at 120 °C. The 1 : 1 ratio shows a crystallite size that drops from 483 nm at 220 °C to 380 nm at 150 °C. The synthesis temperature has been found to have a profound effect on the crystallite size of the BaTiO_3_ samples with a lower synthesis temperature producing crystallites of a smaller size, in agreement with previous work.^15–17^ The BaTiO_3_ samples synthesised using Ba/Ti precursor ratios of 4 : 1 and 2 : 1 exhibit crystallites of smaller sizes, comparing to that using a Ba/Ti precursor ratio of 1 : 1, as a low Ba precursor concentration would result in a smaller amount of BaTiO_3_ nuclei generated in the initial stage of the reaction and a larger crystallite size, again in agreement with previous work.^25^

The tetragonality variation of BaTiO_3_ against the reaction temperature in hydrothermal synthesis with Ba/Ti precursor ratios of 4 : 1, 2 : 1 and 1 : 1 is presented in Fig. 9c. Similar to the trend observed in Fig. 1c, the increasing c/*a* value indicates that the tetragonality of the crystal structure increases with the increasing synthesis temperature in all three Ba/Ti precursor ratios.

**Fig. 10:**
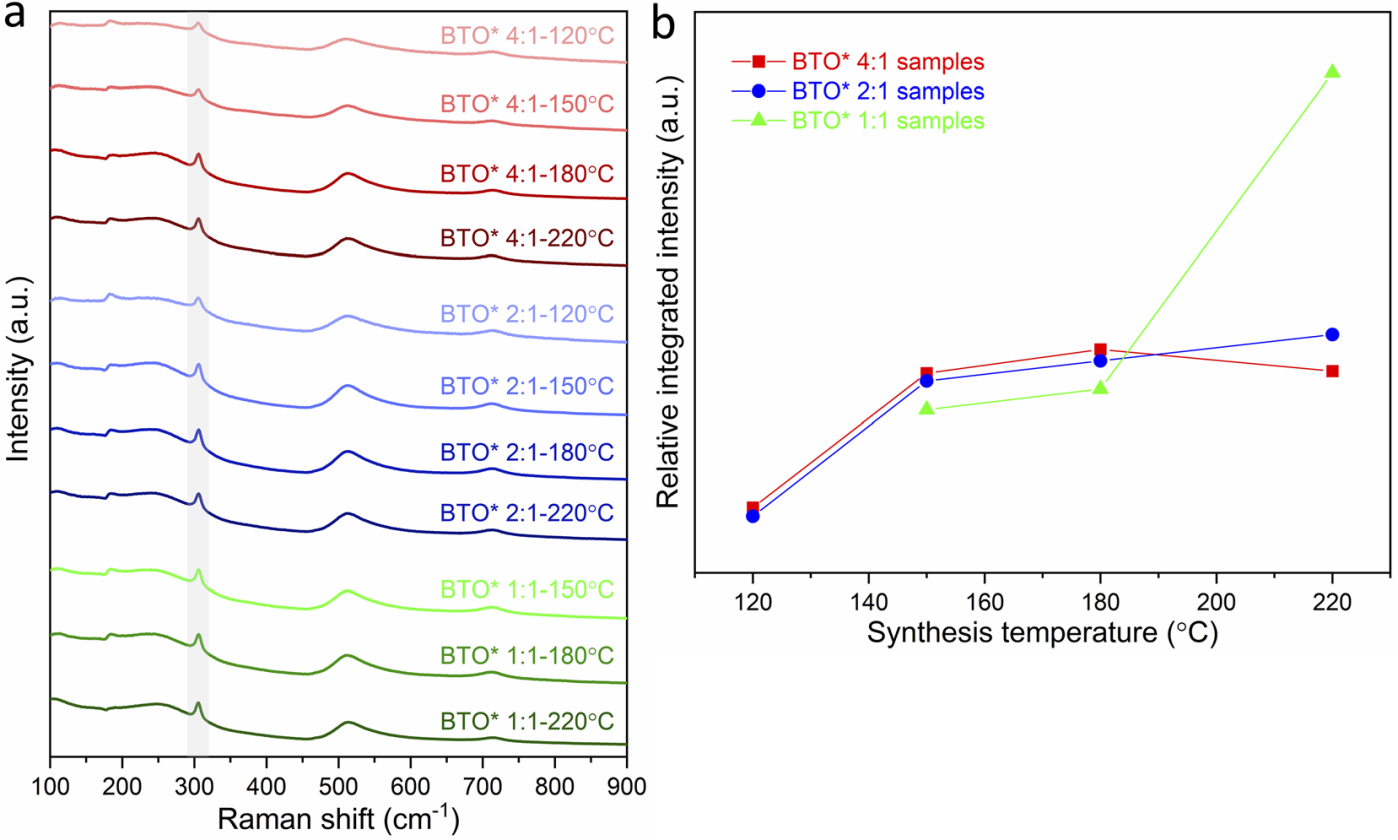
(a) Raman spectra of BaTiO_3_ samples hydrothermally synthesised using Ba/Ti precursor ratios of 4 : 1, 2 : 1 and 1 : 1 with Ba precursor concentration kept constant at 0.09 mol L^−1^, and reacted at various synthesis temperatures of 120, 150, 180 and 220 °C (peaks at ∼307 cm^−1^ being marked as shaded); (b) relative integrated intensities of the Raman peak at 307 cm^−1^ for BaTiO_3_ samples plotted against reaction temperature in hydrothermal synthesis using Ba/Ti precursor ratios of 4 : 1, 2 : 1 and 1 : 1 (sample labels explained in Table 4).

The changes in tetragonality of hydrothermally synthesized BaTiO_3_ samples can also be confirmed by Raman characterisation (Fig. 10a). The variation of the relative integrated intensity of the peak at 307 cm^−1^ for BaTiO_3_*versus* the reaction temperature in hydrothermal synthesis with Ba/Ti precursor ratios of 4 : 1, 2 : 1 and 1 : 1 is presented in Fig. 10b. Similar to the trend observed in Fig. 2b, the increasing integrated intensity of the peak at 307 cm^−1^ indicates that the tetragonality of the crystal structure increases with the increasing synthesis temperature in all three Ba/Ti precursor ratios. This trend agrees with the result obtained from the Rietveld fits to the XRD patterns.

Finally, in order to elucidate the effect of the reagent concentration, the results for the same Ba/Ti precursor ratio, but different reagent concentrations, are shown in Fig. S5.† This figure is obtained by combining the experimental results for the set of conditions in Tables 1 and 4, for the Ba/Ti precursor ratio of 4 : 1 and 1 : 1. When increasing the Ba and Ti precursor concentrations, the crystallite size of BaTiO_3_ shows an overall decrease for samples made by using the Ba/Ti precursor ratio of 4 : 1; however, the BaTiO_3_ crystallite size increases for samples using the Ba/Ti precursor ratio of 1 : 1. Comparing the effect of the precursor concentration (Fig. S5†) and the reaction temperature (Fig. 1b and 9b) on the crystallite size, it is seen that the effect of the reaction temperature is more pronounced.

Hydrothermal synthesis to produce BaTiO_3_ nanoparticles has been investigated by many researchers, and it has been reported that nanosized particles of BaTiO_3_ can be achieved by controlling the reaction conditions.^4^ In our work, nanocrystalline BaTiO_3_ has been prepared by hydrothermal synthesis using barium hydroxide octahydrate and titanium isopropoxide as precursors. The same precursors have been used by Zeng *et al.* to prepared nanocrystalline BaTiO_3_ at low temperature (<100 °C), and the effects of the Ba/Ti precursor ratio and reaction temperature on the size and morphology of BaTiO_3_ were investigated.^10,11^ The same precursors have also been applied by Nahm *et al.* in hydrothermal synthesis to prepare nanocrystalline BaTiO_3_, but they only employed one reaction temperature (220 °C) and Ba/Ti precursor ratio (4 : 1).^12^ In this work, we systematically explored the effects of the reaction temperature (80, 120, 150, 180 and 220 °C) and Ba/Ti precursor ratio (4 : 1, 2 : 1 and 1 : 1) in hydrothermal synthesis for preparing nanocrystalline BaTiO_3_, and we characterised the reaction product by XRD, Raman, SEM, TEM, DLS, TGA and WDS to identify the optimal hydrothermal synthesis conditions in terms of small particle size and low concentration of defects.

## 4. Conclusion

Nanocrystalline BaTiO_3_ has been prepared *via* a hydrothermal synthesis. Reaction conditions including synthesis temperature and Ba/Ti precursor ratio have been controlled and adapted with the aim of producing small crystallites of BaTiO_3_, as well as to investigate the effects of hydrothermal synthesis conditions on the microstructure of BaTiO_3_. The XRD, SEM, TEM and DLS results show that the crystallite/particle size of the BaTiO_3_ samples increases with the increasing synthesis temperature; and the crystallite/particle size of BaTiO_3_ is also affected by the Ba/Ti precursor ratio. The BaTiO_3_ sample synthesised using a Ba/Ti precursor ratio of 2 : 1 at a reaction temperature of 120 °C exhibited homogeneous crystallites of the smallest size of 107 nm. Moreover, the Raman and XRD results indicate that the tetragonality of the crystal structure increases with the increasing synthesis temperature. In addition, the TGA results suggest that the number of defects in BaTiO_3_ decreases as the synthesis temperature increases; and the WDS results indicate that the BaTiO_3_ with less defects was obtained when using a Ba/Ti precursor ratio of 2 : 1.

## Conflicts of interest

There are no conflicts to declare.

## Supplementary Material

RA-012-D2RA03707F-s001

## References

[cit1] Jiang B., Iocozzia J., Zhao L., Zhang H., Harn Y. W., Chen Y., Lin Z. (2019). Chem. Soc. Rev..

[cit2] Kwei G. H., Lawson A. C., Billinge S. J. L. (1993). J. Phys. Chem..

[cit3] West A. R. , Solid State Chemistry and its Applications, John Wiley & Sons Ltd, United Kingdom, 2014

[cit4] Hu M. Z.-C., Kurian V., Payzant E. A., Rawn C. J., Hunt R. D. (2000). Powder Technol..

[cit5] Pithan C., Hennings D., Waser R. (2005). Int. J. Appl. Ceram. Technol..

[cit6] Wegmann M., Brönnimann R., Clemens F., Graule T. (2007). Sens. Actuators, A.

[cit7] Pinceloup P., Courtois C., Leriche A., Thierry B. (1999). J. Am. Ceram. Soc..

[cit8] Zhang M., Fop S., Kramer D., Garcia-Araez N., Hector A. L. (2022). J. Mater. Chem. A.

[cit9] Yun H. S., Yun B. G., Shin S. Y., Jeong D. Y., Cho N. H. (2021). Nanomater.

[cit10] Zeng M. (2011). Appl. Surf. Sci..

[cit11] Zeng M., Uekawa N., Kojima T., Kakegawa K. (2011). J. Mater. Res..

[cit12] Han J.-M., Joung M.-R., Kim J.-S., Lee Y.-S., Nahm S., Choi Y.-K., Paik J.-H., Suvaci E. (2014). J. Am. Ceram. Soc..

[cit13] Larson A., Von Dreele R., Finger L., Kroeker M., Toby B. (2001). J. Appl. Crystallogr..

[cit14] Habib A., Haubner R., Stelzer N. (2008). Mater. Sci. Eng., B.

[cit15] Chen H.-J., Chen Y.-W. (2003). Ind. Eng. Chem. Res..

[cit16] Hennings D., Rosenstein G., Schreinemacher H. (1991). J. Eur. Ceram. Soc..

[cit17] Kumazawa H., Annen S., Sada E. (1995). J. Mater. Sci..

[cit18] Smith M. B., Page K., Siegrist T., Redmond P. L., Walter E. C., Seshadri R., Brus L. E., Steigerwald M. L. (2008). J. Am. Chem. Soc..

[cit19] Wada S., Suzuki T., Noma T. (1995). J. Ceram. Soc. Jpn..

[cit20] El Marssi M., Le Marrec F., Lukyanchuk I. A., Karkut M. G. (2003). J. Appl. Phys..

[cit21] Shiratori Y., Pithan C., Dornseiffer J., Waser R. (2007). J. Raman Spectrosc..

[cit22] Mansuri A., Bhatti I. N., Bhatti I. N., Mishra A. (2018). J. Adv. Dielectr..

[cit23] Shi E.-W., Xia C.-T., Zhong W.-Z., Wang B.-G., Feng C.-D. (1997). J. Am. Ceram. Soc..

[cit24] Ciftci E., Rahaman M. N., Shumsky M. (2001). J. Mater. Sci..

[cit25] Eckert J. O., Hung-Houston C. C., Gersten B. L., Lencka M. M., Riman R. E. (1996). J. Am. Ceram. Soc..

