# Supplementary material for: Effects of the reaction temperature and Ba/Ti precursor ratio on the crystallite size of BaTiO3 in hydrothermal synthesis
Source: RSC Adv. 2022 Sep 29;12(43):27809–19. doi: 10.1039/d2ra03707f (PMC9520380; doi:10.1039/d2ra03707f)
Supplement: RA-012-D2RA03707F-s001 [file RA-012-D2RA03707F-s001.pdf]

Electronic Supplementary Material (ESI) for RSC Advances.
This journal is © The Royal Society of Chemistry 2022

Supplementary Information

Effects of the reaction temperature and Ba/Ti precursor ratio on the crystallite size of $BaTiO_3$ in hydrothermal synthesis

Min Zhang,[a] *Joseph Falvey,*[a] *Andrew L. Hector **[a] *and Nuria Garcia-Araez* [a,b]

[a] School of Chemistry, University of Southampton, Highfield, Southampton SO17 1BJ, UK
E-mail: A.L.Hector@soton.ac.uk

[b] The Faraday Institution, Quad One, Harwell Campus, Didcot OX11 0RA, UK

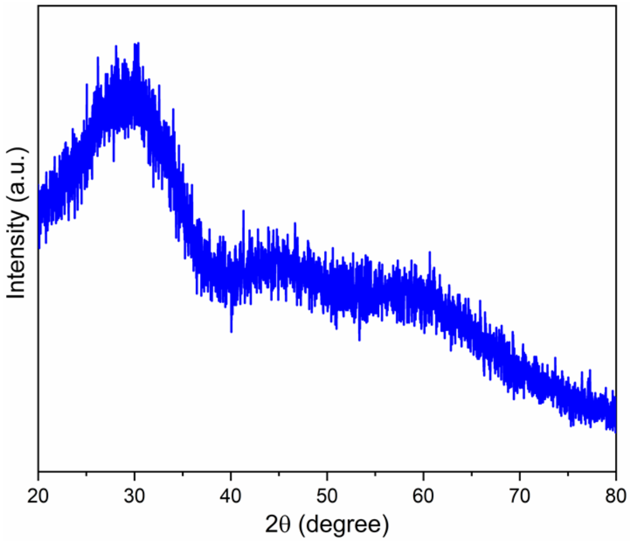

Fig. S1 XRD pattern of amorphous TiO_2 powder precipitated when using $Ti(OCH(CH_3)_2)_4$ as the Ti precursor in hydrothermal synthesis of $BaTiO_3$.

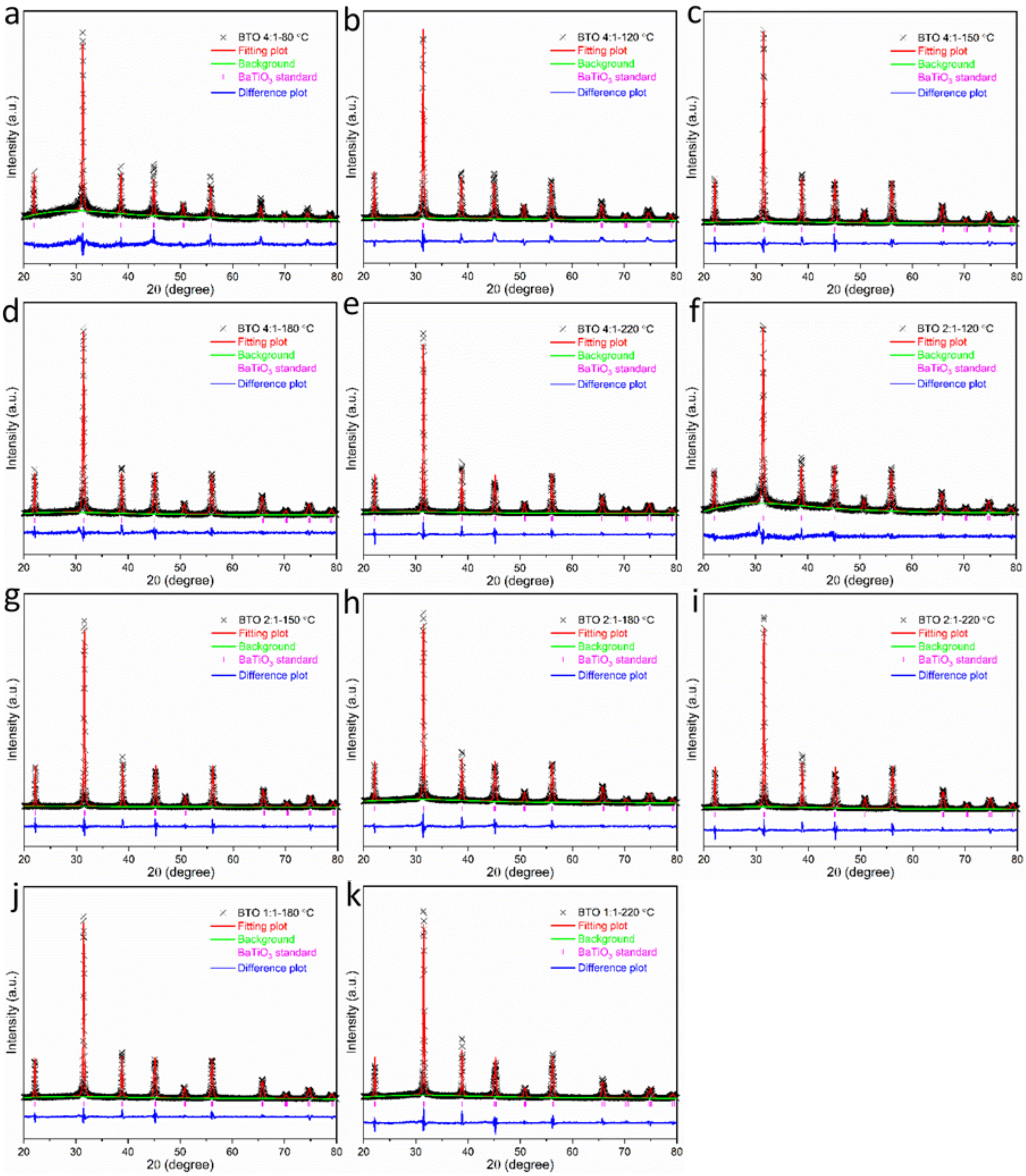

Fig. S2 Rietveld fits to the XRD patterns of (a) BTO 4:1-80 °C, (b) BTO 4:1-120 °C, (c) BTO 4:1-150 °C, (d) BTO 4:1-180 °C, (e) BTO 4:1-220 °C, (f) BTO 2:1-120 °C, (g) BTO 2:1-150 °C, (h) BTO 2:1-180 °C, (i) BTO 2:1-220 °C, (j) BTO 1:1-180 °C and (k) BTO 1:1-220 °C samples (sample labels explained in Table 1). The data points and Rietveld fits are overlaid as black crosses and red lines, respectively. The difference plots and background are shown in blue and green, respectively. The pink tick marks represent the allowed reflection positions for tetragonal $BaTiO_3$ structure with space group *P4mm*.

Table S1 Lattice parameters (a, c), tetragonality (c/a), Lorentzian values (L_x, L_y) and reliability factors (R_{wp}, R_p) obtained from the Rietveld fits to the XRD patterns of $BaTiO_3$ samples (sample labels explained in Table 1).

Sample	Lattice parameter		Tetragonality	Lorentzian value		Reliability factor	
	a [Å]	c [Å]	c/a	L_x	L_y	R_{wp}	R_p
BTO 4:1-80 °C	4.0308(4)	4.0304(9)	0.9999	6.15(24)	33.09	0.1478	0.1197
BTO 4:1-120 °C	4.0153(2)	4.0285(3)	1.0033	3.89(15)	48.78	0.0975	0.0778
BTO 4:1-150 °C	4.0105(2)	4.0276(2)	1.0043	3.60(10)	38.68	0.1044	0.0807
BTO 4:1-180 °C	4.0100(3)	4.0251(4)	1.0038	3.42(17)	47.88	0.1312	0.0973
BTO 4:1-220 °C	4.0044(1)	4.0244(2)	1.0050	2.22(9)	34.76	0.1102	0.0836
BTO 2:1-120 °C	4.0101(4)	4.0242(6)	1.0035	7.37(26)	44.37	0.1157	0.0883
BTO 2:1-150 °C	4.0073(2)	4.0236(2)	1.0041	3.20(11)	32.98	0.1385	0.1061
BTO 2:1-180 °C	4.0070(2)	4.0233(2)	1.0041	3.13(12)	38.40	0.1074	0.0849
BTO 2:1-220 °C	4.0049(1)	4.0232(2)	1.0046	2.26(9)	36.00	0.1048	0.0811
BTO 1:1-180 °C	4.0085(1)	4.0241(2)	1.0039	2.36(10)	38.95	0.1004	0.0778
BTO 1:1-220 °C	4.0007(2)	4.0255(2)	1.0062	2.12(10)	32.83	0.1097	0.0871

Note that L_x is Lorentzian broadening associated with the crystallite size, and L_y is Lorentzian broadening associated with the microstrain.

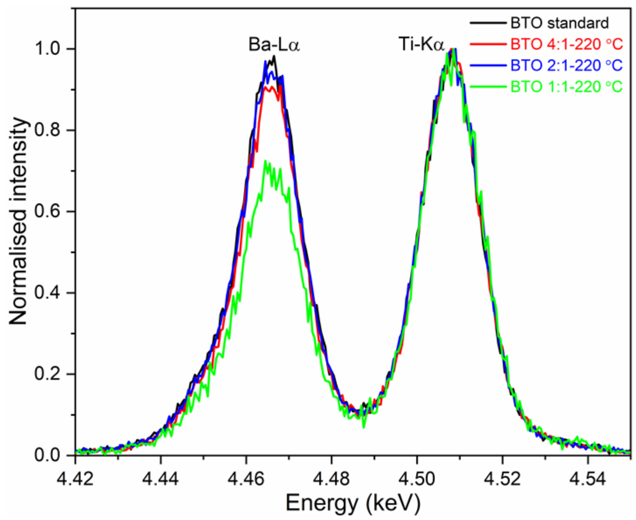

Fig. S3 WDS spectra of the standard $BaTiO_3$ and $BaTiO_3$ samples hydrothermally synthesised at 220 °C using Ba/Ti precursor ratios of 4:1, 2:1 and 1:1 (sample labels explained in Table 1).

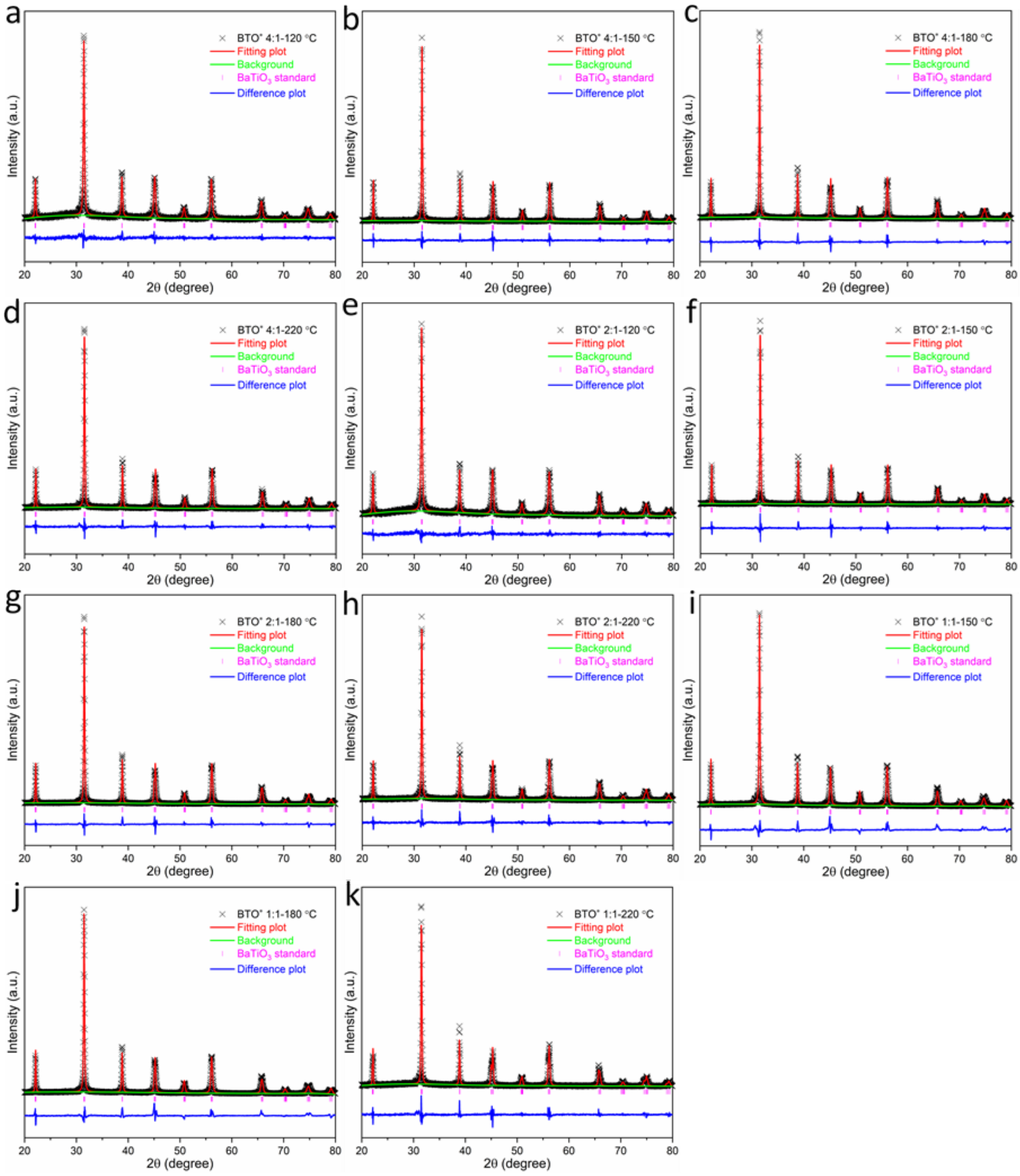

Fig. S4 Rietveld fits to the XRD patterns of (a) BTO* 4:1-120 °C, (b) BTO* 4:1-150 °C, (c) BTO* 4:1-180 °C, (d) BTO* 4:1-220 °C, (e) BTO* 2:1-120 °C, (f) BTO* 2:1-150 °C, (g) BTO* 2:1-180 °C, (h) BTO* 2:1-220 °C, (i) BTO* 1:1-150 °C, (j) BTO* 1:1-180 °C and (k) BTO* 1:1-220 °C samples (sample labels explained in Table 4). The data points and Rietveld fits are overlaid as black crosses and red lines, respectively. The difference plots and background are shown in blue and green, respectively. The pink tick marks represent the allowed reflection positions for tetragonal $BaTiO_3$ structure with space group *P4mm*.

Table S2 Lattice parameters (a, c), tetragonality (c/a), Lorentzian values (L_x, L_y) and reliability factors (R_{wp}, R_p) obtained from the Rietveld fits to the XRD patterns of $BaTiO_3$ samples (sample labels explained in Table 4).

Sample	Lattice parameter		Tetragonality	Lorentzian value		Reliability factor	
	a [Å]	c [Å]	c/a	L_x	L_y	R_{wp}	R_p
BTO* 4:1-120 °C	4.0104(3)	4.0251(4)	1.0037	5.05(20)	47.74	0.1176	0.0893
BTO* 4:1-150 °C	4.0083(2)	4.0231(2)	1.0037	2.45(11)	31.37	0.1396	0.1060
BTO* 4:1-180 °C	4.0045(1)	4.0230(2)	1.0046	2.33(9)	31.38	0.1110	0.0867
BTO* 4:1-220 °C	4.0065(2)	4.0241(3)	1.0044	2.00(12)	36.14	0.1444	0.1097
BTO* 2:1-120 °C	4.0089(3)	4.0239(4)	1.0037	7.02(19)	40.18	0.1182	0.0893
BTO* 2:1-150 °C	4.0076(2)	4.0242(2)	1.0041	2.60(10)	30.27	0.1460	0.1118
BTO* 2:1-180 °C	4.0057(1)	4.0237(2)	1.0045	2.53(9)	33.36	0.1093	0.0850
BTO* 2:1-220 °C	4.0047(2)	4.0225(2)	1.0044	2.04(12)	34.59	0.1418	0.1060
BTO* 1:1-150 °C	4.0110(3)	4.0221(4)	1.0028	2.07(15)	44.63	0.1596	0.1221
BTO* 1:1-180 °C	4.0075(2)	4.0223(3)	1.0037	1.77(14)	43.44	0.1455	0.1109
BTO* 1:1-220 °C	4.0010(2)	4.0266(2)	1.0064	1.63(12)	30.66	0.1509	0.1195

Note that L_x is Lorentzian broadening associated with the crystallite size, and L_y is Lorentzian broadening associated with the microstrain.

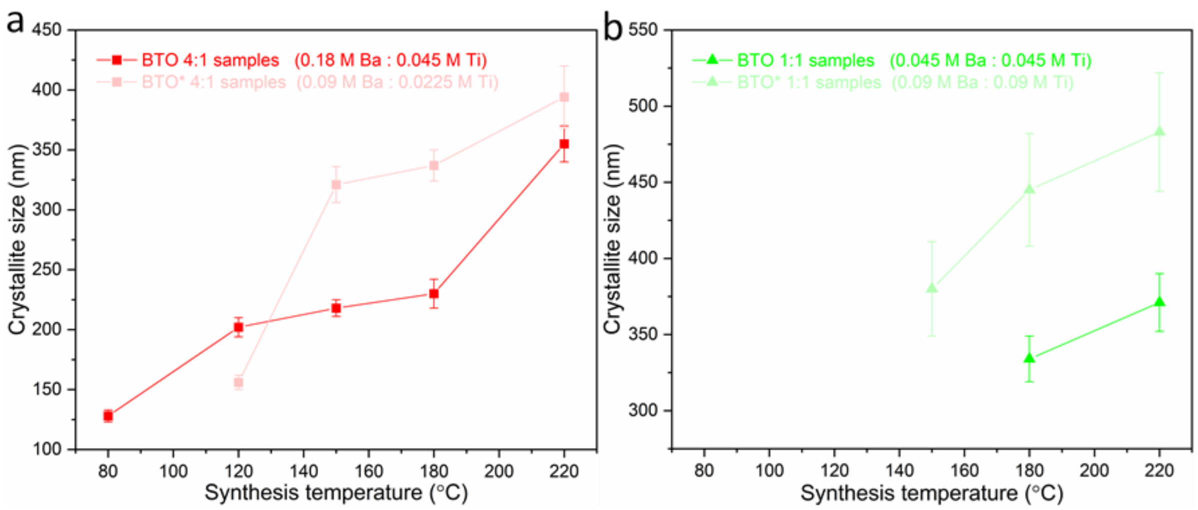

Fig. S5 Crystallite size of $BaTiO_3$ samples plotted against reaction temperature in hydrothermal synthesis using Ba/Ti precursor ratios of (a) 4:1 and (b) 1:1, obtained from the Rietveld fits to the XRD patterns, for the reaction conditions of (a) 0.18 M Ba : 0.045 M Ti and 0.09 M Ba : 0.0225 M Ti, (b) 0.045 M Ba : 0.045 M Ti and 0.09 M Ba : 0.09 M Ti. Sample labels explained in Table 1 and Table 4.
